# ACCESS TO HIGH-QUALITY HOME HEALTH CARE FOR PERSONS WITH DEMENTIA

**DOI:** 10.1093/geroni/igae098.2154

**Published:** 2024-12-31

**Authors:** Harriet Mather

**Affiliations:** Icahn School of Medicine at Mount Sinai, New York, New York, United States

## Abstract

While prior research has demonstrated variation in access to high quality home health agencies among older Medicare beneficiaries, access among persons with dementia - a population with increased need of skilled care delivered in the home - has not been explored. This study examined availability of high-quality home health agencies by dementia prevalence in the US using a cross-sectional analysis of county-level data on availability and quality of home health agencies indexed to number of FFS Medicare beneficiaries with Alzheimer’s disease and related dementia (ADRD). Using multiple Medicare Public Use Files (PUF), including star ratings for home health quality of patient care (QoPC), home health care CAHPS survey (HHCAHPS), county-level ADRD prevalence, and Medicare FFS enrollment, we found that the median county-level number of home health agencies was 15 (IQR 8-29), star rating was 3.4 (IQR 3.1-3.7) for QoPC and 3.8 (IQR 3.5-4.0) for HHCAHPS. Higher prevalence of persons with ADRD was moderately correlated with total number of home health agencies in a county (Spearman’s r=0.36, p<0.05), with weaker positive correlations with high-quality home health agencies via QoPC (r=.2, p<0.05) and HHCAHPS quality ratings (r=.17, p<0.05). There is wide variation in access to high quality home health care among persons with dementia. To meet the needs of a growing population living at home with dementia, we must address inequities in access to high quality home health care.

